# Comparative outcomes of video-assisted thyroidectomy and traditional open surgery: a 5-year analysis of a single center experience

**DOI:** 10.1016/j.bjorl.2024.101539

**Published:** 2024-12-12

**Authors:** Kenzo Ohara, Takumi Kumai, Kan Kishibe, Hidekiyo Yamaki, Hiroki Komatsuda, Tatsuya Hayashi, Miki Takahara

**Affiliations:** Asahikawa Medical University, Department of Otolaryngology-Head and Neck Surgery, Asahikawa, Japan

**Keywords:** Endoscopic thyroid surgery, Video-assisted neck surgery, Lobectomy, Thyroid tumor, Gasless lifting retractor

## Abstract

**Objective:**

This study aimed to evaluate and compare the outcomes of patients undergoing video-assisted thyroidectomy versus those undergoing traditional open surgery.

**Methods:**

In this retrospective study, we reviewed the clinical records of 449 patients who underwent thyroid surgery at our institution between April 2017 and March 2022. Of these, 209 underwent video-assisted thyroidectomy, while 248 underwent traditional open surgery. After excluding those who received total thyroidectomy, central node dissection, or lateral node dissection, we focused our analysis on 136 patients with video-assisted lobectomy and 92 patients with traditional open surgery. We compared patient outcomes, including surgical results and adverse events, between these groups.

**Results:**

Significant differences were observed in the maximum tumor diameter between the video-assisted lobectomy and traditional open surgery groups. However, no significant differences were noted in surgical time, bleeding, or adverse events.

**Conclusion:**

Video-Assisted Neck Surgery (VANS) offers great cosmetic benefits and is comparable to traditional open surgery in terms of adverse events.

**Level of evidence:**

IV.

## Highlights

• We compared the outcomes of patients undergoing VANS versus open lobectomy.
• No significant differences were observed in adverse events between two groups.
• Our VANS technique could be a good option in thyroid lobectomy.

## Introduction

Thyroid nodules exhibit a higher prevalence in women compared to men, with an increase in occurrence observed with advancing age. The initial introduction of the VANS technique, using two pieces of Kirschner wire to enhance the surgical view, was pioneered by Shimizu in 1998.^1^ At our institution, VANS utilizing a subclavian approach has been the preferred method for thyroid surgery since 2009, primarily aimed at minimizing neck scarring. There are several minimal invasive techniques for endoscopic thyroidectomy, such as endoscopic transoral thyroidectomy^2^, ^3^, ^4^, ^5^ and endoscopic transaxillary thyroidectomy,^6^, ^7^ both of which effectively avoid visible anterior neck scars. However, at our institution, the risk of injury to the inferior dental nerve in endoscopic transoral thyroidectomy, along with the necessity of using longer forceps compared to open surgery in transaxillary thyroidectomy, pose significant challenges. The VANS technique is familiar to head and neck surgeons due to its direct organ manipulation capability, without the need for Carbon Dioxide (CO_2_) gas insufflation.^1^, ^8^, ^9^ However, one of the challenges associated with VANS is the adaptation period required for surgeons to become proficient with the narrower view and the intricacies of single-port procedures.

In the field of gastrointestinal surgery, surgeons often progress to single-port laparoscopic surgery after gaining proficiency in multiport techniques. In head and neck surgery, due to the spatial constraints, surgeons are often required to adapt to single-port endoscopic surgery, as creating multiport operating conditions is not feasible.^10^ Herein, we aimed to compare the outcomes of VANS with those of Traditional Open Surgery (TOS), evaluating if there are significant differences in patient results.

## Methods

### Patients

Between April 2017 and March 2019, a total of 449 patients underwent thyroid surgeries at our institution. Of these, 209 underwent endoscopic thyroidectomy using the VANS method, while 248 underwent TOS. We excluded patients who underwent either partial or total thyroidectomy, as well as those who underwent central or lateral node dissection. Between the VANS and TOS groups, we retrospectively reviewed patient outcomes, including surgical results and adverse events.

The study was approved by the Institutional Review Board of our institution (approval number C2242), and all participants provided written informed consent before treatment initiation. This study adhered to the principles of the Declaration of Helsinki.

### Study protocol

Thyroid tumors were evaluated using palpation, ultrasound scans, and computed tomography. Flexible laryngoscopy was performed preoperatively to assess the integrity of the recurrent laryngeal nerves. Fine needle aspiration cytology was conducted in all cases. The indication for VANS lobectomy were as follows: preoperatively diagnosed benign tumors with a longitudinal diameter of less than 50 mm by the end of 2020, and less than 80 mm since the beginning of 2021.

### Operative procedures

**Fig. 1:**
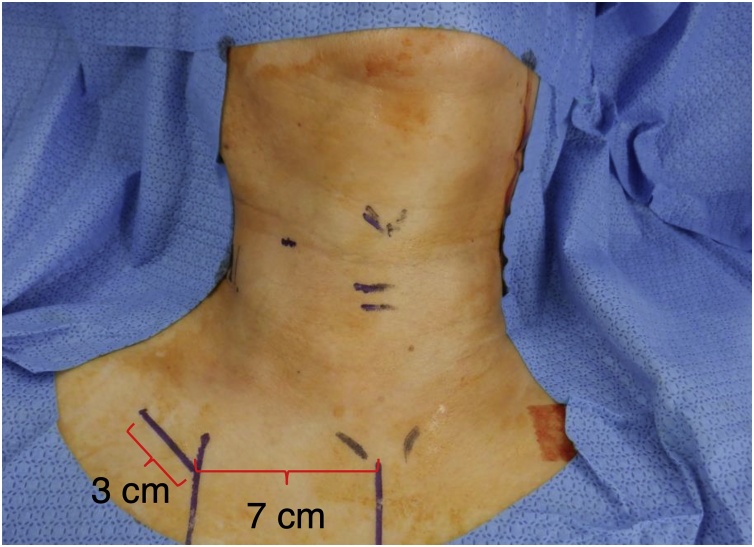
Skin incision site. A 30 mm skin incision was made in the subclavian area, located 7 cm from sternal notch.

**Fig. 2:**
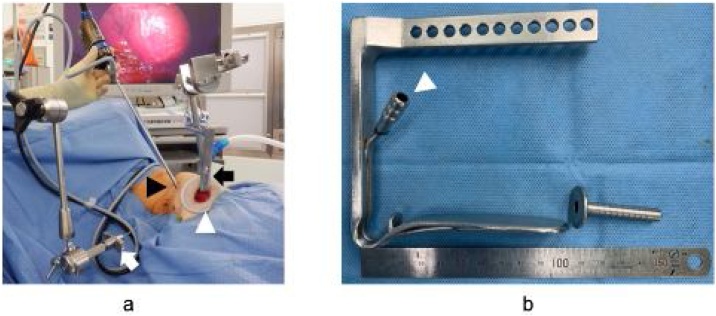
Surgical view and instruments. (a) Macro view of VANS thyroid surgery, showing the placement of the Lap Protector™ (white arrow-head) at the subclavian wound. A gasless lifting retractor (black arrow) was used to enhance the surgical view. A 5-mm camera port (black arrow-head) was centered on the gasless lifting retractor. A muscle retractor (white arrow) was used to retract the sternocleidomastoid and sternohyoid muscles. (b) A gasless lifting retractor with an aspirator (white arrow-head) and a 5-mm camera port.

**Fig. 3:**
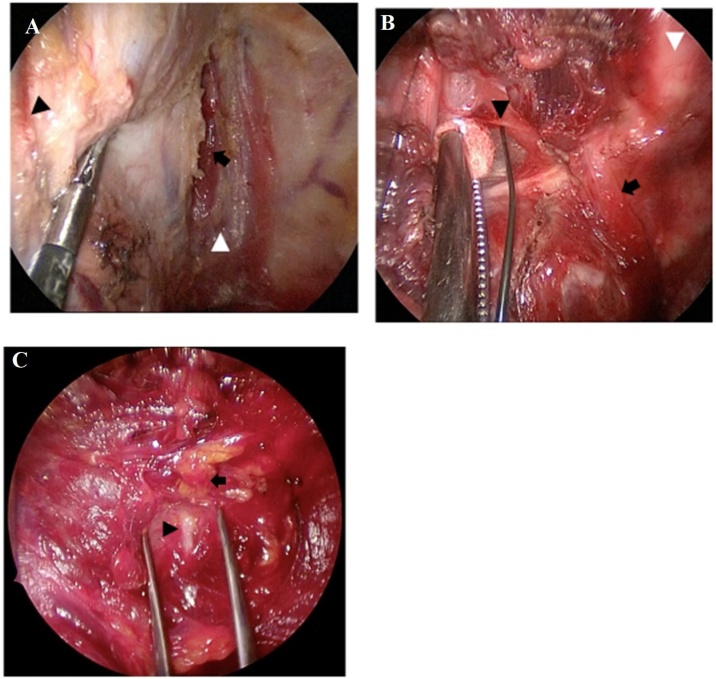
Operative procedures. (a) Central approach technique for accessing the thyroid lobe. A white arrowhead indicates the midline, and a black arrow indicates the right sternohyoid muscle. The right sternocleidomastoid muscle was retracted to left (black arrowhead). (b) The right external branch of superior laryngeal nerve (black arrowhead) was identified by nerve integrity monitor. The black arrow indicates the right cricothyroid muscle. The white arrowhead indicates the trachea. (c) The right RLN (black arrowhead) was identified near the right superior parathyroid (black arrow).

**Fig. 4:**
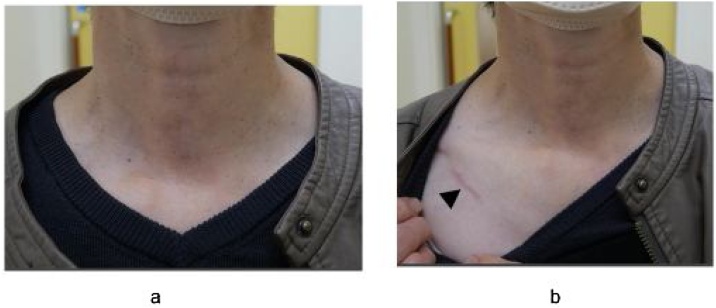
Postoperative wounds. (a and b) The subclavian wound (black arrowhead) along the skin crease is concealed under regular clothing. The images show a patient one year after undergoing right thyroid lobectomy via the VANS method.

A 30 mm incision along the skin crease, located 7 cm from the sternal notch, was made in the affected subclavian area (Fig. 1). The subplatysmal skin flap was elevated sufficiently to place a gasless lifting retractor with an aspirator. The Lap ProtectorTM was used to protect the wound before retractor placement. A 5-mm camera port was centrally positioned on the gasless lifting retractor. The sternocleidomastoid and sternohyoid muscles were retracted using a muscle retractor to access the thyroid lobe (Fig. 2a and b). We employed a central approach to expose the thyroid gland, which involved dividing the sternohyoid muscle at the center for a better surgical view (Fig. 3a). After adequately exposing the superior thyroid pole, the external branch of the superior laryngeal nerve was identified in the avascular space between the cricothyroid and upper pole using a nerve integrity monitor (Fig. 3b). The superior thyroid artery and vein were ligated using an energy device such as LigaSureTM, Harmonic®, or BiClamp®. Employing the capsular dissection technique,^11^, ^12^ we dissected the middle thyroid veins and inferior thyroid vessels prior to encountering the Recurrent Laryngeal Nerve (RLN). The superior parathyroid gland was preserved after detection, enabling easier localization of the RLN around the level of the cricoid cartilage, situated behind the plane of the parathyroid gland (Fig. 3c).^13^ The thyroid gland was then separated from Berry’s ligament and the trachea, and the lobectomy completed with isthmus transection. Surgical wounds were not visible under regular clothing (Fig. 4a and b).

### Statistical analyses

All statistical analyses were conducted using GraphPad Prism version 10 (GraphPad Software, San Diego, CA, USA). The clinical characteristics of the patients were compared between the VANS group and the TOS group using Chi-Square test, Mann-Whitney *U* test and Fisher’s exact test. Multivariate analysis was performed utilizing multiple and logistic regression analyses.

## Results

**Fig. 5:**
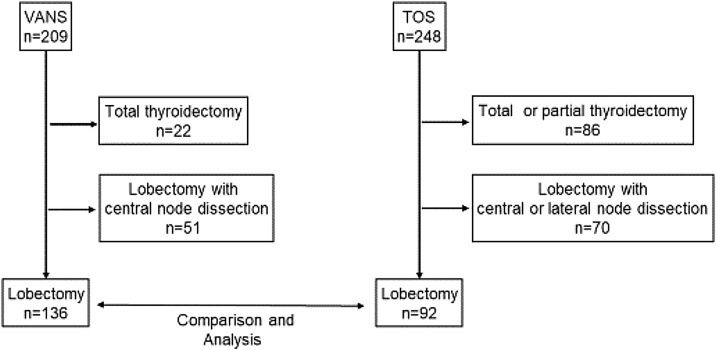
Study protocol flow chart. This flow chart details the inclusion and exclusion of patients in the study.

**Fig. 6:**
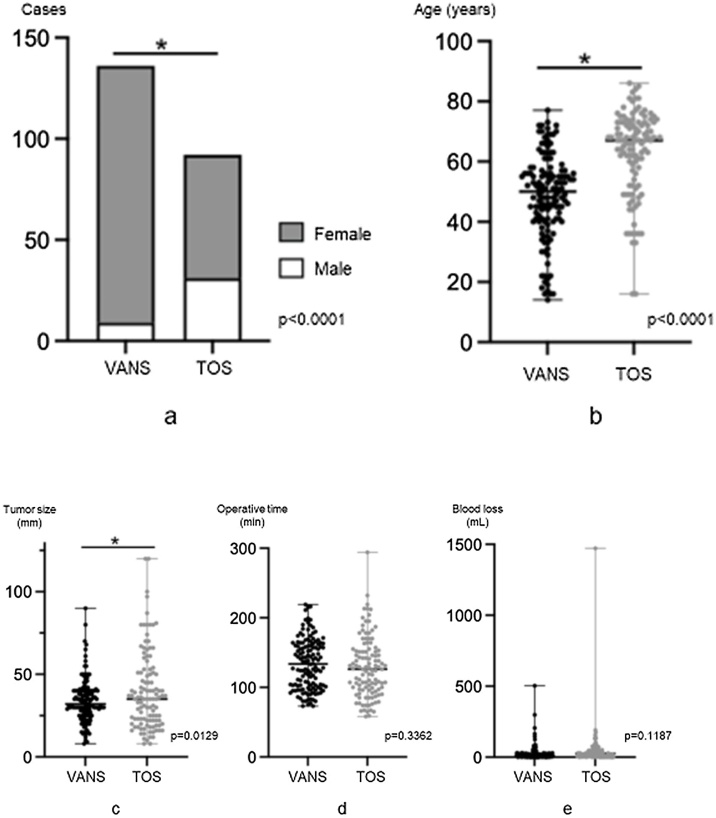
Statistical analyses. (a) Differences in sex ratio between the VANS and TOS groups. (b) Differences in age between the VANS and TOS groups. (c) Differences in tumor size between the VANS and TOS groups. (d) Differences in operative time between the VANS and TOS groups. (e) Differences in blood loss between the VANS and TOS groups.

After applying exclusion criteria, we ultimately included 136 patients who underwent lobectomy using the VANS method and 92 patients who underwent TOS (Fig. 5). In the VANS group, there were 127 female patients, while the TOS group comprised 61 female patients. Female patients were significantly more likely to undergo thyroidectomy via the VANS method (Chi-Square test: *p* <  0.0001) (Fig. 6a). The mean age of patients in the VANS group was 48.4 years (range 14–77 years; median 50 years), compared to 62.95 years (range 16–86 years; median 67 years) in the TOS group, indicating a statistically significant difference (Mann-Whitney *U* test: *p* <  0.0001) (Fig. 6b), suggesting that younger patients tend to opt for the VANS technique. The mean maximum tumor diameters were 33.9 mm (range 8–90 mm; median 32 mm) in the VANS group and 39.9 mm (range 8–120 mm; median 35 mm) in the TOS group, also showing a significant difference (Mann-Whitney *U* test: *p* =  0.0129) (Fig. 6c). This discrepancy is attributed to our preference for TOS in tumors exceeding 50 mm before 2020 and 80 mm thereafter. Regarding operative time, no significant difference was observed between the two groups (Fig. 6d). Similarly, blood loss during surgery did not significantly differ between the groups (Fig. 6e).

**Fig. 7:**
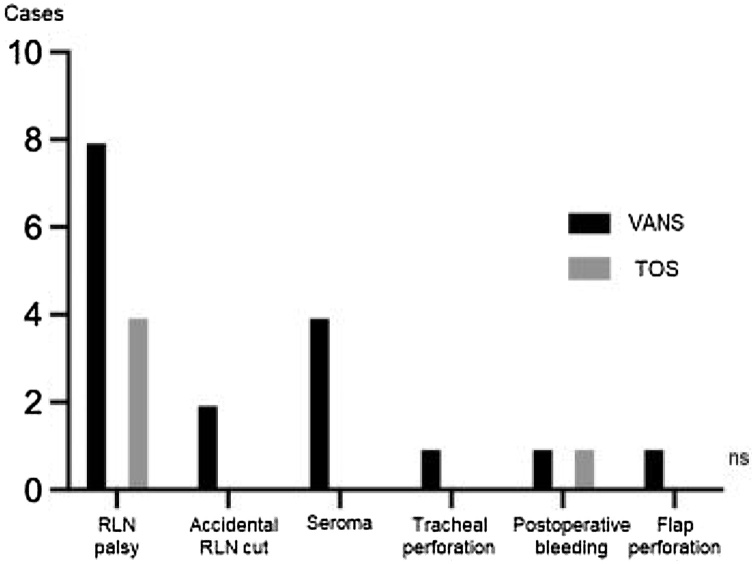
Postoperative complications. Differences in RLN palsy, accidental RLN cut, seroma, tracheal perforation, postoperative bleeding and flap perforation between the groups.

Postoperative complications, including RLN palsy, accidental RLN transection, seroma, tracheal perforation, postoperative bleeding, and flap perforation, did not significantly differ between the VANS and TOS groups, as evaluated using Fisher’s exact test (Fig. 7). Specifically, for RLN palsy, no significant correlation was found with operative time, sex, affected side, or tumor size by multivariate analysis. However, the right side exhibited a trend (*p* =  0.0719) towards a higher occurrence of RLN palsy compared to other factors (Table 1). We encountered two cases of accidental RLN transection, but immediate reconstructions were performed in these cases, resulting in improved hoarseness within three months post-surgery.

**Table 1:** Multivariate analysis of predictors of RLN palsy in VANS group.

Independent variables	Estimated regression coefficient	Standard error	95% CI (asymptotic)	*t*-value	*p*-value
Operative time	0.0003	0.0005	−0.0006580 to 0.001186	0.5662	0.5722
Sex	−0.0723	0.0873	−0.2450 to 0.1004	0.828	0.4092
Affected side (right)	0.0783	0.0432	−0.007080 to 0.1637	1.814	0.0719
Size	0.0004	0.0015	−0.002644 to 0.003367	0.2381	0.8122

In our study, none of the patients required conversion from VANS to TOS. The tumor size was a predictor of longer operative time, as expected (Table 2).

**Table 2:** Multivariate analysis of predictors of operative time in VANS group.

Independent variables	Estimated regression coefficient	Standard error	95% CI (asymptotic)	*t*-value	*p*-value
Sex	22.4400	16.1900	−9.585 to 54.46	1.386	0.1681
Affected side (right)	13.7700	7.9730	−2.002 to 29.54	1.727	0.0865
Size	1.7400	0.2400	1.265 to 2.215	7.251	<0.0001

## Discussion

Our study aimed to compare the surgical outcomes of patients undergoing VANS with those undergoing TOS for thyroidectomy. Our findings indicate that while VANS offers a significant cosmetic advantage, it does not differ significantly from TOS in terms of surgical duration, blood loss, and postoperative complications.

Since Shimizu’s introduction of VANS in 1998,^1^ we have developed an innovative gasless lifting retractor with an aspirator, which replaces the use of wires and offers a clearer surgical view without the issue of fogging. A notable advantage of VANS is the ability to use hands, to some extent, through the main port under the subclavian area, allowing for tactile identification of the thyroid lobe or trachea. Moreover, the forceps in VANS are only marginally longer than those in TOS and the surgical view closely resembles that of TOS, making it more accessible for head and neck surgeons. Consequently, we have favored VANS over other minimal invasive endoscopic thyroidectomy techniques. However, manual knot-tying is challenging in the narrow surgical field, necessitating the use of energy devices for dissecting thyroid vessels. While these devices facilitate VANS, surgeons must consider potential thermal injury to the RLN.^14^ Although intraoperative neuromonitoring has not shown a significant decrease in RLN palsy during thyroid surgery,^15^ its use is recommended in VANS. This is not only for RLN identification but also to mitigate the risk of unexpected RLN palsy in the constrained surgical field. In our institution, we employ a superior parathyroid gland approach for RLN identification.^13^ Typically, the superior parathyroid gland is located at the level of the cricoid cartilage.^16^ Furthermore, as the superior parathyroid gland is situated between the true and false capsules of the thyroid gland, we can identify the RLN while dissecting the false capsule from the caudal-to-cranial side around the superior parathyroid.^12^, ^13^ In the VANS method, operators and assistants can simultaneously view the surgical field, offering an enlarged and shared view. This feature is advantageous not only for its cosmetic benefits but also for educational purposes. It allows trainee doctors can learn how to identify RLN not only during surgery but also through pre- and post-operative review of recorded videos.

Our study found a higher likelihood of RLN palsy on the right side compared to the left (Table 1). This may be attributed to the anatomical differences, as the right RLN crosses the inferior thyroid artery more obliquely and is more laterally oriented,^17^ leading to potentially stronger tissue traction during capsular dissection on the right side, resulting in a higher incidence of RLN palsy.^18^

A disadvantage of VANS approach is the restricted surgical space it offers. Despite providing a close view, particularly around the RLN, the overall surgical field is narrower than that in TOS. However, with increased familiarity with this constrained view, endoscopic thyroid surgery could become the gold standard for thyroid lobectomy, similar to laparoscopic cholecystectomy.^19^ In this study, we focused exclusively on benign thyroid tumors, which were relatively large (median 32 mm, average 33.9 mm). The operative time in the VANS group tended to increase with tumor size (Table 2). Future analyses should include a broader range of VANS cases, incorporating malignant tumors as well.

A limitation of this study was the relatively small number of patients involved. Another constraint was the fewer number of operators in the VANS group compared to the TOS group. The proficiency required for VANS, necessitating comprehensive knowledge and technical skill in thyroid surgery, meant that all nine VANS operators in this study had to be certified in otolaryngology and head and neck surgery by the national society of otorhinolaryngology head and neck surgery. Notably, one surgeon performed 69 out of the 136 VANS cases, whereas the TOS group comprised 23 surgeons. This underlines the need to train more doctors in the VANS method to promote its global adoption. Given that VANS is not yet a common approach in thyroid surgery, trainee doctors are advised to first learn traditional open thyroidectomy. Consequently, the number of operators in the TOS group was larger, including 13 trainee doctors out of 23 surgeons. Additionally, the tumor sizes in the TOS group were significantly larger than those in the VANS group (Fig. 5c). These factors may have contributed to the lack of significant differences in operative time and postoperative complications between the VANS and TOS groups.

## Conclusion

VANS offers superior cosmetic results compared to TOS. There were no significant differences in the rate of complications between the VANS and TOS groups. Although VANS is not currently the first-choice method for thyroid surgery, it has the potential to become a feasible option, similar to laparoscopic cholecystectomy, in the foreseeable future.

## Ethical consideration

All procedures performed in this studies involving human participants follows the 1964 Helsinki declaration and its later amendments or comparable ethical standards. We obtained written informed consent from all individual participants included in the study.

## Funding

The authors did not receive any specific funding for this study.

## Conflicts of interest

The authors declare no conflicts of interest.

## References

[1] Shimizu K., Akira S., Tanaka S. (1998). Video-assisted neck surgery: endoscopic resection of benign thyroid tumor aiming at scarless surgery on the neck. J Surg Oncol.

[2] Wang C., Zhai H., Liu W., Li J., Yang J., Hu Y. (2014). Thyroidectomy: a novel endoscopic oral vestibular approach. Surgery.

[3] Lira R.B., De Cicco R., Rangel L.G., Bertelli A.A., Duque Silva G., de Medeiros Vanderlei J.P. (2021). Transoral endoscopic thyroidectomy vestibular approach: experience from a multicenter national group with 412 patients. Head Neck.

[4] Deshmukh P., Shiva B., Yadav S.K., Agarwal P., Sharma D., Johri G. (2024). A comparison of swallowing related quality of life in patients undergoing transoral endoscopic versus open thyroid surgery. World J Surg.

[5] Kasemsiri P., Trakulkajornsak S., Bamroong P., Mahawerawat K., Piromchai P., Ratanaanekchai T. (2020). Comparison of quality of life between patients undergoing trans-oral endoscopic thyroid surgery and conventional open surgery. BMC Surg.

[6] Hyun K., Byon W., Park H.J., Park Y., Park C., Yun J.S. (2014). Comparison of swallowing disorder following gasless transaxillary endoscopic thyroidectomy versus conventional open thyroidectomy. Surg Endosc.

[7] Jasaitis K., Midlenko A., Bekenova A., Ignatavicius P., Gulbinas A., Dauksa A. (2021). Transaxillary gasless endoscopic thyroidectomy versus conventional open thyroidectomy: systematic review and meta-analysis. Wideochir Inne Tech Maloinwazyjne.

[8] Hegedüs L. (2004). Clinical practice. The thyroid nodule. N Engl J Med.

[9] Shimizu K., Kitagawa W., Akasu H., Hatori N., Hirai K., Tanaka S. (2002). Video-assisted endoscopic thyroid and parathyroid surgery using a gasless method of anterior neck skin lifting: a review of 130 cases. Surg Today.

[10] Lin H., Zhang J., Li X., Li Y., Su S. (2023). Comparative outcomes of single-incision laparoscopic, mini-laparoscopic, four-port laparoscopic, three-port laparoscopic, and single-incision robotic cholecystectomy: a systematic review and network meta-analysis. Updates Surg.

[11] Delbridge L. (2003). Total thyroidectomy: the evolution of surgical technique. ANZ J Surg.

[12] Tan Y.H., Du G.N., Xiao Y.G., Guo S.Q., Wu T., Chen P.Z. (2013). The false thyroid capsule: new findings. J Laryngol Otol.

[13] Elsheikh E. (2017). Superior parathyroid gland approach to the recurrent laryngeal nerve. Head Neck.

[14] Wang J.J., Huang T.Y., Wu C.W., Lin Y.C., Tseng H.Y., Liu C.H. (2021). Improving voice outcomes after thyroid surgery - review of safety parameters for using energy-based devices near the recurrent laryngeal nerve. Front Endocrinol.

[15] Higgins T.S., Gupta R., Ketcham A.S., Sataloff R.T., Wadsworth J.T., Sinacori J.T. (2011). Recurrent laryngeal nerve monitoring versus identification alone on post-thyroidectomy true vocal fold palsy: a meta-analysis. Laryngoscope.

[16] Serpell J.W. (2010). New operative surgical concept of two fascial layers enveloping the recurrent laryngeal nerve. Ann Surg Oncol.

[17] Makay O., Icoz G., Yilmaz M., Akyildiz M., Yetkin E. (2008). The recurrent laryngeal nerve and the inferior thyroid artery-anatomical variations during surgery. Langenbecks Arch Surg.

[18] Chiang F.Y., Lu I.C., Kuo W.R., Lee K.W., Chang N.C., Wu C.W. (2008). The mechanism of recurrent laryngeal nerve injury during thyroid surgery ‒ the application of intraoperative neuromonitoring. Surgery.

[19] Kim S.S., Donahue T.R. (2018). Laparoscopic cholecystectomy. JAMA.

